# An Investigation Into the Yields of 3,4‐Methylenedioxyphenyl‐2‐propanone (MDP2P) From Glycidate Pre‐Precursors

**DOI:** 10.1002/dta.70143

**Published:** 2026-09-07

**Authors:** Alexandra L. Mercieca, Ella S. Schuett, Andrew M. McDonagh, Shanlin Fu, Morgan Alonzo

**Affiliations:** ^1^ School of Mathematical and Physical Sciences University of Technology Sydney Sydney New South Wales Australia; ^2^ Centre for Forensic Science, School of Mathematical and Physical Sciences University of Technology Sydney Sydney New South Wales Australia

**Keywords:** EMDMG, glycidates, MDMA, MDP2P, MMDMG, pre‐precursors

## Abstract

The use of pre‐precursors to illicitly manufacture 3,4‐methylenedioxyphenyl‐2‐propanone (MDP2P) for the synthesis of 3,4‐methylenedioxymethylamphetamine (MDMA) has emerged in clandestine laboratories. Methyl 3‐(3,4‐methylenedioxyphenyl)‐2‐methyl glycidate (MMDMG) and ethyl 3‐(3,4‐methylenedioxyphenyl)‐2‐methyl glycidate (EMDMG) have recently been seized in clandestine laboratories in New South Wales, Australia. These glycidate esters can be converted to MDP2P via acid or base hydrolysis. This study investigated factors influencing MDP2P yield from MMDMG and EMDMG through both pathways. For acid hydrolysis, hydrochloric, sulphuric, phosphoric and acetic acids were examined at different reaction times and temperatures. Reactions at 100°C produced higher MDP2P yields than those at room temperature, and 18 h was sufficient for complete conversion. Hydrochloric and sulphuric acids gave the highest yields after 18 h at 100°C (79% and 78%, respectively), phosphoric acid gave moderate conversion (~30%), and acetic acid produced poor yields (< 10%). The conversion proceeded via two previously unreported diol‐ester intermediates. Base hydrolysis involved heating glycidate esters with sodium hydroxide in alcohol, forming sodium piperonyl methyl ketone (PMK) glycidate, followed by reaction with aqueous acid. Heating MMDMG in methanol or EMDMG in ethanol for 5 h produced sodium PMK glycidate. This intermediate was unstable in acidic aqueous solution and rapidly converted to MDP2P at room temperature. Yields obtained using hydrochloric, sulphuric and phosphoric acids (73%, 71% and 69%, respectively) were comparable to acid hydrolysis, whereas acetic acid produced lower yields (25%). This work may assist law enforcement agencies to predict illicit drug yields from pre‐precursors and support investigations into emerging illicit drug synthesis trends.

## Graphical abstract

Hydrolysis of the glycidate pre‐precursors MMDMG and EMDMG was evaluated for MDP2P production. Acid hydrolysis of MMDMG (HCl, 100°C, 18 h) yielded the highest MDP2P recovery (79%), whereas base hydrolysis produced only slightly lower yields (73%), demonstrating both pathways are effective routes to this key MDMA precursor.

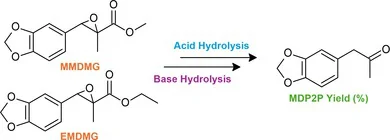

## 1 Introduction

Illicit drug pre‐precursors are attractive synthetic starting materials for 3,4‐methylenedioxyphenyl‐2‐propanone (MDP2P) and phenyl‐2‐propanone (P2P) for clandestine synthesis of 3,4‐methylenedioxymethylamphetamine (MDMA) and methylamphetamine, respectively. Precursors used in traditional syntheses such as pseudoephedrine, safrole and piperonal are controlled and restricted, and so clandestine production of precursors P2P and MDP2P from pre‐precursors provides a method to circumvent such controls.

The phenomenon of designer pre‐precursors is constantly evolving as restrictions on existing precursors are implemented. Designer pre‐precursors can be ‘masked’ with additional functional groups to evade detection and circumvent legislation. Common pre‐precursors include α‐phenylacetoacetonitrile (APAAN), methyl α‐phenylacetoacetate (MAPA), methyl α‐acetyl‐(3,4‐methylenedioxyphenyl)acetate (MAMDPA), isopropylidene (2‐(3,4‐methylenedioxyphenyl)acetyl)malonate (IMDPAM), α‐phenylacetoacetamide (APAA) and diethyl (phenylacetyl)propanedioate (DEPAPD) [1, 2, 3, 4]. These pre‐precursors are readily converted using simple acid or base hydrolysis methods to yield P2P or MDP2P precursors in various yields. As many of these pre‐precursors come under international control, clandestine operators are prompted to seek alternatives.

The synthesis of P2P and MDP2P from glycidic acid and glycidic ester pre‐precursors provides alternative synthetic routes to these compounds. Glycidic acids and glycidic esters can be converted into ketones or aldehydes using hydrolysis methods [5]. Acid hydrolysis has been observed in clandestine laboratories using a one‐pot procedure. Base hydrolysis is a two‐step process whereby a sodium or potassium glycidate is first obtained by de‐esterification using alcoholic sodium or potassium hydroxide. The glycidate salt can be converted to the corresponding ketone by pyrolysis [5, 6], or by reaction with aqueous acid [5, 7, 8] or heating copper powder [9, 10]. Some common glycidate and glycidic esters that have emerged in clandestine operations include methyl 3‐(3,4‐methylenedioxyphenyl)‐2‐methyl glycidate (MMDMG) and ethyl 3‐(3,4‐methylenedioxyphenyl)‐2‐methyl glycidate (EMDMG) for MDP2P production and P2P methyl glycidic acid and its esters for P2P production.

This work examined MMDMG and EMDMG, Figure 1, that were discovered in clandestine laboratory seizures associated with illicit MDP2P synthesis in New South Wales (NSW), Australia [11]. From an Australian perspective, the most commonly encountered clandestine laboratories in NSW manufacture methamphetamine, followed by MDMA [11]. EMDMG and MMDMG are the most prevalent glycidate‐derived analogues seized in clandestine laboratories [11]. This is consistent with international trends where EMDMG has become the dominant MDMA pre‐precursor reported; in 2024, 6280 t of EMDMG in MDMA equivalents were seized [12]. Previous work [10] investigated some seized material and characterised a mixture of *cis* and *trans* isomers of MMDMG and examined one method of conversion to MDP2P. However, across clandestine operations, it is reported that different experimental conditions are employed including the various reaction times, temperatures and acids employed for hydrolysis. No work to date has reported on the factors that influence the yields of MDP2P from either MMDMG or EMDMG.

**FIGURE 1:**
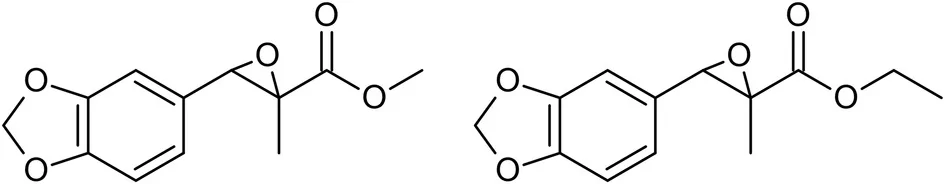
Structure of MMDMG (left) and EMDMG (right).

In the current work, we examined seized EMDMG and MMDMG and the conversion of these compounds to MDP2P. Two methods for the conversion of MMDMG and EMDMG to MDP2P were investigated, as shown in Scheme 1. Method 1 involved acid hydrolysis of the glycidate esters by heating in acid. The impact of time, temperature and the type of acid were explored to reflect different reaction conditions observed in clandestine laboratories. Hydrochloric acid, sulphuric acid, phosphoric acid and acetic acid were investigated. Method 2 involved base hydrolysis using alcoholic sodium hydroxide to form MDP2P glycidic acid (as the sodium salt, Method 2a) followed by decarboxylation in aqueous acid (Method 2b). All of the aforementioned acids were investigated for Method 2b. This investigation of the experimental conditions that influence the yields of MDP2P has the potential to assist law enforcement in forensic case work to estimate potential illicit drug yields.

**SCHEME 1:**
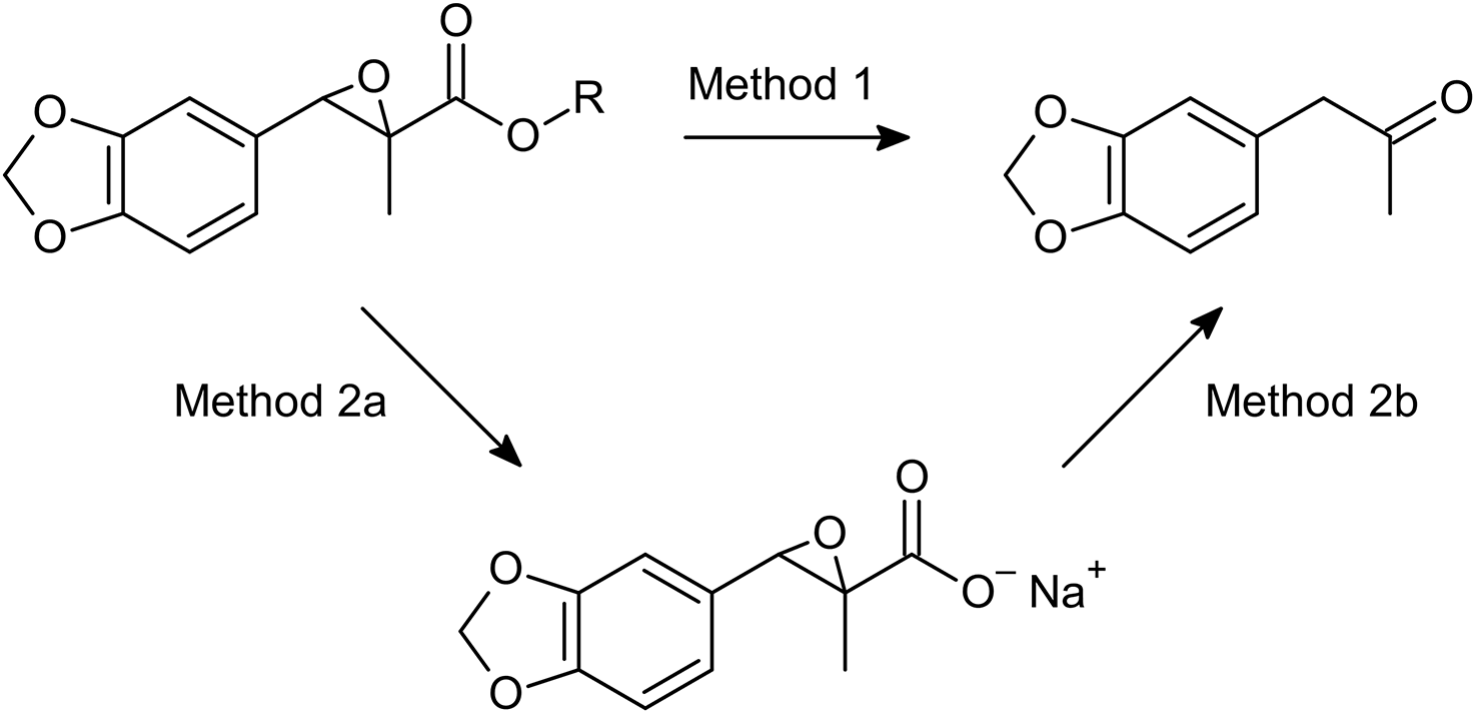
Pathways to MDP2P. R = CH_3_, CH_2_CH_3_.

## 2 Materials and Methods

### 2.1 Chemicals and Reagents

All chemicals were used as received. The following chemicals were purchased from Sigma Aldrich (Melbourne, Victoria, Australia): chloroform‐d (CDCl_3_, 99.8%) containing 0.03% (v/v) tetramethylsilane (TMS), dimethyl sulfoxide‐d6 ((CD_3_)_2_SO, 99.9%), deuterium oxide (99.9 atom % D), benzoic acid (standard for quantitative NMR [qNMR], TraceCERT), acetonitrile (hypergrade for LC–MS), hydrochloric acid (32%), dichloromethane, benzopinacolone (2,2,2‐triphenylacetophenone, 98%). The following chemicals were purchased from Chemsupply (Adelaide, South Australia, Australia): ethyl acetate, hexane, anhydrous sodium sulphate and glacial acetic acid. The following chemicals were purchased from various suppliers: sulphuric acid (98%, RCI Labscan, Bangkok Thailand), phosphoric acid (Ortho, 85%, Rowe Scientific, Wangara, Western Australia, Australia). MMDMG and EMDMG (samples of seized material) were provided by the Drug and Firearms Squad, New South Wales Police Force (NSWPF). MDP2P was synthesised using previous literature procedures [13, 14].

Syntheses for methyl‐3‐(1,3‐benzodioxol‐5‐yl)‐2,3‐dihydroxy‐2‐methylpropanoate and ethyl‐3‐(1,3‐benzodioxol‐5‐yl)‐2,3‐dihydroxy‐2‐methylpropanoate are described in the Supporting Information together with characterisation data (^1^H NMR, ^13^C NMR, GC–MS, HRMS).

### 2.2 Sample Preparation

#### 2.2.1 Stock, Working and Internal Standard (IS) Solutions

Stock solutions of MMDMG, EMDMG and MDP2P were prepared by creating a 10,000 ppm mixed stock solution in acetonitrile. Benzopinacolone was chosen as the IS for the quantification of MMDMG, EMDMG and MDP2P for its chemical stability, reproducible MS response and absence in seized drug samples. The IS was prepared as a 4000 ppm stock solution in ethyl acetate.

Calibration standards and quality control (QC) samples were prepared by adding the appropriate volume of analyte standard and 25.0 μL of IS solution, then diluting to a final volume of 1.0 mL. Concentrations for selected analytes were between 50 and 200 ppm. QC samples were prepared at three concentration levels (low, medium and high) within the validated linear range. The corresponding concentrations were 90, 140 and 180 ppm, respectively. All standards and QC samples were vortex‐mixed and filtered through a 0.45 μm syringe filter prior to analysis.

#### 2.2.2 Reaction Product Samples

For the quantification of analytes using gas chromatography–mass spectrometry (GC–MS), working samples were prepared by dispensing 20.0 μL of the reaction product samples (Section 2.5.1, 2.5.3) and 25.0 μL of IS and 955 μL of acetonitrile. Samples were then vortex‐mixed and filtered through a 0.45 μm syringe filter prior to analysis.

For the qualitative analysis of by‐products using ^1^H nuclear magnetic resonance (NMR) spectroscopy, a 1.0 mL aliquot was reduced under a gentle stream of nitrogen at 60°C until dry. The sample was then reconstituted in CDCl_3_ and vortex‐mixed prior to analysis.

#### 2.2.3 Synthesis Samples

Synthesis samples for GC–MS analysis were prepared by dissolving 10.0 mg of analyte in 1 mL of acetonitrile. A 20.0 μL aliquot was taken and diluted to 1.0 mL (200 ppm), which was then vortex‐mixed and filtered through a 0.45 μm syringe filter prior to analysis. For high‐resolution mass spectrometry, a 100 ppm bridging stock solution was prepared by diluting 10.0 μL of the primary stock solution to 1.00 mL with acetonitrile. A 100.0 μL aliquot was taken from the bridging solution and diluted to 1.0 mL to create a 10 ppm solution. Samples were then vortex‐mixed and filtered through a 0.45 μm syringe filter prior to analysis.

Characterisation of EMDMG, MMDMG, MDP2P and synthesised diol‐esters by ^1^H and ^13^C NMR spectroscopy was performed by dissolving approximately 10.0 mg of each sample in 600 μL of CDCl_3_.

### 2.3 Instrumentation

#### 2.3.1 GC–MS

GC–MS was performed using an Agilent 7890A series gas chromatographic system coupled to an Agilent 5975C Network Mass Selective Detector. Analytes were separated using an Agilent DB5‐MS column (30 m × 0.25 mm × 0.25 μm). Helium was the carrier gas with a flow rate of 1.0 mL/min. The injection temperature was set to 280°C and employed a split injection in a 25:1 ratio with 1 μL injections. The oven temperature was initially set at 60°C and held for 3 min, followed by a ramp of 25°C/min to 300°C, where it was held for 5 min. A 3.3 min solvent delay was applied, and the total run time was 17.6 min. Full‐scan mass spectra were collected within the scan range of *m/z* 30–500 for identification of by‐products. Selected ion monitoring (SIM) was employed for quantification of MMDMG, EMDMG and MDP2P. Qualifier ions, quantifier ions and retention times are listed in Table 1. Data was processed and analysed using the GC/MSD ChemStation software (Version 01.03.2357). Mass spectra and retention times of MMDMG, EMDMG, MDP2P and both diol‐esters are provided in Figures S1–S4. Total ion chromatograms (TICs) from all hydrolysis experiments are provided in Figures S5–S33.

**TABLE 1:** Retention times, qualifier ions and quantifier ions used in the selected ion monitoring of analytes by GC–MS.

Analyte	RT (min)	Qualifier ions (*m/z*)	Quantifier ion (*m/z*)
Benzopinacolone (IS)	13.692	165, 243	243
EMDMG	10.298	149, 179, 250	250
MMDMG	10.021	149, 165, 236	236
MDP2P	8.549	135, 178	178

Abbreviations: IS = internal standard, RT = retention time.

#### 2.3.2 NMR Spectroscopy

NMR spectra were recorded using a Bruker Ascend (400.17 MHz) or Bruker Ascend Evo (400.27 MHz) spectrometer and data were processed using Bruker Topspin software (Version 4.1.3). Purity of synthesised MDP2P was > 95% by qNMR spectroscopy. Sample preparation, processing of NMR spectra and calculation of purity for MDP2P, seized MMDMG and EMDMG, and synthesised diol‐esters were performed according to recommended guidelines [15]. Sample preparation procedure and spectroscopy parameters are provided in Table S1. Chemical shifts (δ) are reported in ppm and the spectra calibrated according to the residual solvent chemical shift (^1^H NMR; CDCl_3_ = 7.26 ppm, (CD_3_)_2_SO = 2.50 ppm, ^13^C NMR; CDCl_3_ = 77.16 ppm, (CD_3_)_2_SO = 39.52). ^1^H and ^13^C NMR spectra of MMDMG, EMDMG and both diol‐esters are provided in Figures S34–S41.

#### 2.3.3 High‐Resolution Mass Spectrometry

High‐resolution mass spectrometry was performed using an Agilent 1290 Infinity LC system coupled to an Agilent 6510 qTOF mass spectrometer. The mass spectrometer was equipped with an electrospray ionisation (ESI) source operating in positive mode and tuned prior to every run. Optimised source parameters were as follows: gas temperature 275°C; drying gas 10 L/min; nebuliser pressure 25 psig; fragmentor voltage 100 V; skimmer voltage 60 V; capillary voltage 3500 V. For each analysis, 0.5 μL of sample was injected using flow injection analysis at a flow rate of 0.25 mL/min using water and methanol as mobile phases in a 10:90 (v/v) ratio.

#### 2.3.4 Polarimetry

Optical rotation measurements were recorded using a JASCO P‐2000 polarimeter equipped with a sodium lamp (589 nm at 25°C) using a 1 dm quartz cell. Samples were dissolved in ethyl acetate to the required concentrations (*c*) reported in g/100 mL.

### 2.4 Method Validation

The fit‐for‐purpose method was validated for MDP2P, MMDMG and EMDMG using GC–MS in accordance with internationally recognised analytical validation guidelines [16, 17, 18]. The method was validated for the following parameters: selectivity, working range, limit of detection (LOD) and limit of quantification (LOQ), accuracy and precision, ruggedness and stability in storage.

Selectivity was evaluated by analysing samples from different sources to ensure the absence of interfering signals during analysis. These included blank samples, samples containing only the target analytes, samples containing only the IS and reaction product samples. Each sample was analysed in six replicates. Acceptable selectivity was defined as the absence of interfering signals in the extracted ion chromatogram at the quantifier ion. Linearity was evaluated from seven‐point IS calibration curves constructed by plotting the peak area ratio of the analyte to the IS against corresponding concentrations (50–200 ppm). LOD and LOQ were determined based on the standard deviation of the response from blank samples. Ten blank samples were injected, and the standard deviation (S_0_) of the response was calculated. LOD was calculated as $\mathrm{LOD}=\frac{\left(3.3\times S_{0}\right)}{m}$ and LOQ as $\mathrm{LOQ}=\frac{\left(10\times S_{0}\right)}{m}$, where *m* is the gradient of the calibration curve. Precision and accuracy were evaluated using QC samples of low, medium and high concentrations (90, 140, 180 ppm). Intra‐day (within‐day) and inter‐day (between‐day) variability were used to assess precision by analysing seven replicates of each level over 2 weeks. Precision and accuracy were evaluated using the percentage of coefficient of variation (%CV) and percentage mean relative error (%MRE), respectively. For medium and high QC samples, CV ≤ 15% and MRE within ±15% were considered acceptable. For low QC samples, CV ≤ 20% CV and MRE within ±20% were considered acceptable. Ruggedness was evaluated by introducing minor deliberate variations to the analytical method to determine whether quantification remained consistent. Three replicates were analysed under modified conditions, including small changes in inlet temperature, temperature ramp rate and initial oven temperature. Performance was evaluated against the original method based on accuracy (±15% MRE) and precision (≤ 15% CV) requirements. Sample stability during storage was evaluated by re‐injecting low and high QC samples and reaction product samples (*n* = 3) following 1 week of storage at 6°C. Stability was considered acceptable if the measured concentrations were within ±15% MRE and had a CV ≤ 15% relative to freshly prepared samples.

### 2.5 MDP2P Synthesis From Glycidate Compounds

#### 2.5.1 Method 1

MMDMG (100 mg, 0.42 mmol) or EMDMG (100 mg, 0.40 mmol) was dissolved in water (5 mL) and heated at 25°C or 100°C in aqueous acid (1.0 mL, 2 mol excess) for a measured period of time. The reaction was then cooled and organic components extracted into dichloromethane (3 × 10 mL) and washed with water and saturated aqueous sodium chloride. The organic portion was then dried over sodium sulphate, filtered and the solvent removed. The residue was then dissolved in 10 mL of acetonitrile using sonication. The reaction product solution was stored at 6°C until analysis.

#### 2.5.2 Method 2a

MMDMG (100 mg, 0.42 mmol) or EMDMG (100 mg, 0.40 mmol) was heated at reflux in a solution of sodium hydroxide (30 mg, 0.75 mmol) in 90% aqueous alcohol (methanol for MMDMG and ethanol for EMDMG, 5 mL) for 5 h. The solvent was then removed on a rotary evaporator.

#### 2.5.3 Method 2b

Water (5 mL) was added to the residue from Method 2a and aqueous acid in excess (3 M equivalents based on glycidate) was added and the reaction mixture stirred at room temperature for 10 min. The organic components were extracted into dichloromethane (3 × 10 mL) and washed with water and saturated aqueous sodium chloride. The organic portion was then dried over sodium sulphate, filtered and the solvent removed.

The residue was then dissolved in 10 mL of acetonitrile using sonication. The reaction product solution was stored at 6°C until analysis.

## 3 Results and Discussion

### 3.1 Seized Glycidate Characterisation

MMDMG and EMDMG, supplied by the NSWPF, were analysed by qNMR and found to be 97% and 98% pure, respectively. ^1^H and ^13^C NMR spectra of the seized MMDMG and EMDMG contained only one set of signals for each compound (Figures S34–S37). That is, only one isomer was present in each sample. The ^1^H NMR chemical shifts in the spectrum of MMDMG are consistent with reported data [10, 19] and corresponded to the *trans* isomer. This material is somewhat different from that reported previously [10] in which the seized MMDMG contained a mixture of *cis* and *trans* isomers (with the *trans* isomer the major component). The ^1^H NMR spectrum acquired for EMDMG was similar to that of MMDMG (with the exception of the signals assigned to the ethyl protons) and indicated that only one isomer was present, which we assign to the *trans* isomer. The synthesis of MMDMG or EMDMG from piperonal using the method of Elks and Hey [9] has been reported to form a mixture of *cis* and *trans* isomers [10]. The identification of only the *trans* isomer in the seized material suggests either an alternative synthetic route was used or that the products were purified by some means. The impact of these stereoisomers on the yields of MDP2P may be interesting to explore in future work.

GC–MS in full scan mode identified no other compounds in the starting material. The mass spectrum of MMDMG is consistent with previous reports [10] and showed a molecular ion of *m/z* 236 and base peak of *m/z* 149. The mass spectrum of EMDMG differs slightly from previous characterisation spectra for the compound whereby a base peak of *m/z* 179 was reported [20]. However, we reported a base peak of *m/z* 149.

Polarimetry experiments revealed [*a*]_*D*_ values of −0.17 and +0.62 for MMDMG and EMDMG, respectively. These data indicate that both seized materials are racemic (within experimental error).

### 3.2 Method Validation

No interfering peaks were observed in the extracted ion chromatograms, indicating the method was selective for MDP2P, MMDMG and EMDMG. All analytes displayed acceptable linearity (*R* ^2^ > 0.99) over the concentration range of 50–200 ppm. Table 2 summarises LOD, LOQ, accuracy and precision data, with acceptable values obtained for MMDMG, EMDMG and MDP2P. The method was evaluated as rugged as minor changes to the starting temperature, inlet temperature and ramp rate did not impact accuracy and precision. All analytes were stable under storage conditions (6°C) for up to 1 week (Table S2). The recovery of MMDMG, EMDMG and MDP2P was also within the acceptable limits (Table S3).

**TABLE 2:** Method validation parameters for analytes, including limit of detection (LOD), limit of quantification (LOQ), and accuracy (%MRE) and intra‐ and inter‐day precision (%CV) at low, medium and high quality control levels.

Analyte	Analytical limits (ppm)		Accuracy (%MRE) (*n* = 7)			Intra‐day precision (%CV) (*n* = 7)			Inter‐day precision (%CV) (*n* = 14)		
	LOD	LOQ	Low^a^	Med	High	Low	Med	High	Low	Med	High
MMDMG	0.001	0.004	5.8	2.3	0.9	2.5	3.2	6.2	2.5	2.8	4.6
EMDMG	0.003	0.009	6.2	1.9	1.7	2.2	4.3	5.7	2.7	3.1	4.1
MDP2P	0.008	0.024	2.4	1.3	2.1	2.2	5.2	6.3	2.1	3.6	4.6

^a^ Low = 90 ppm, med = 140 ppm, high 180 ppm.

### 3.3 Acid Hydrolysis of MMDMG and EMDMG (Method 1)

Data for the acid hydrolysis reactions of MMDMG and EMDMG are presented in Table 3.

**TABLE 3:** Acid hydrolysis (Method 1) reaction conditions and corresponding yields of MDP2P from MMDMG and EMDMG starting material.

Entry	Starting compound	Temp (°C)	Acid_(aq)_	Time (h)	Yield of MDP2P^a^
1	MMDMG	25	Nil^d^	3	Below LOD
2		100	Nil	18	Below LOQ^b^
3		25	HCl	3	Below LOD
4		100	HCl	3	55%
5		100	HCl	6	78%
6		100	HCl	18	79%
7		100	H_2_SO_4_	3	44%
8		100	H_2_SO_4_	18	78%^c^
9		100	H_3_PO_4_	3	2%
10		100	H_3_PO_4_	18	34%
11		100	CH_3_COOH	3	Below LOD
12		100	CH_3_COOH	18	9%
13	EMDMG	25	Nil	3	Below LOD
14		100	Nil	18	Below LOQ^b^
15		25	HCl	3	Below LOD
16		100	HCl	3	42%
17		100	HCl	6	76%
18		100	HCl	18	75%
19		100	H_2_SO_4_	3	40%
20		100	H_2_SO_4_	18	59%^c^
22		100	H_3_PO_4_	3	2%
23		100	H_3_PO_4_	18	29%
24		100	CH_3_COOH	3	Below LOD
25		100	CH_3_COOH	18	9%

^a^ Average internal molar yield (%) calculated from GC–MS data (*n* = 3).

^b^ MDP2P detected; however, in concentrations below calculated LOQ for analyte.

^c^ Black insoluble solid formed during this reaction.

^d^ Water only, no acid.

#### 3.3.1 Effect of Reaction Temperature

Heating MMDMG and EMDMG at 100°C improved the yield of MDP2P compared with room temperature from below limits of detection to 42%–55% (Entries 3 and 4 for MMDMG and 15 and 16 for EMDMG, Table 3). Reaction at room temperature gave unreacted glycidate ester as well as two new diol‐ester intermediates (see below). When heated at 100°C using the same time and acid, MDP2P was identified alongside the diol‐ester intermediates. This suggested that heating accelerates the hydrolysis of the glycidate ester to form the diol‐ester intermediate.

#### 3.3.2 Characterisation of Diol‐Ester Intermediates

The TICs obtained by GC–MS analysis following stirring of MMDMG and EMDMG in aqueous hydrochloric acid at room temperature for 3 h revealed compounds in addition to the glycidate starting material that were not MDP2P (Figures S9 and S10). The mass spectra (Figures S3 and S4) contained base peaks of *m/z* 151 common to the methyl and ethyl analogues, indicative of a structural component common to both compounds. This is similar to a fragment observed with isosafrole glycol, where a hydroxyl group on the α‐benzylic carbon gives a base peak of *m/z* 151 [21]. In addition, both molecular ions were heavier by *m/z* 18 than the glycidate esters, typical of a water molecule.

Analysis of the NMR and GC–MS spectra indicated that these are the new compounds methyl 3‐(1,3‐benzodioxol‐5‐yl)‐2,3‐dihydroxy‐2‐methylpropanoate and ethyl 3‐(1,3‐benzodioxol‐5‐yl)‐2,3‐dihydroxy‐2‐methylpropanoate and are assigned the structures shown in Figure 2. ^1^H and ^13^C NMR spectra of the pure (by qNMR) diol‐esters are provided in Figures S38–S41. Two sets of ^1^H NMR signals were present in both spectra, which we assigned to the two sets of enantiomers (2*R,3S & 2S,3R*) and (*2R,3R & 2S,3S*) associated with the two chiral centres. Stereoisomers were not separated nor was the major isomer determined. Interestingly, the ^1^H NMR spectra acquired using CDCl_3_ as solvent contained signals assigned to the OH protons as well as coupling of the vicinal protons to the adjacent OH protons. Figure S42 (top) shows the relevant region of the ^1^H NMR spectrum of MMDMG. Proton **a** appears as a doublet due to coupling to the OH proton **b** (which correspondingly appears as a doublet). Proton **c**, assigned to the OH group adjacent to the ester groups is observed as a singlet. The assignment of the OH protons was confirmed by addition of D_2_O to the NMR sample, which resulted in deuterium exchange (Figure S42).

**FIGURE 2:**
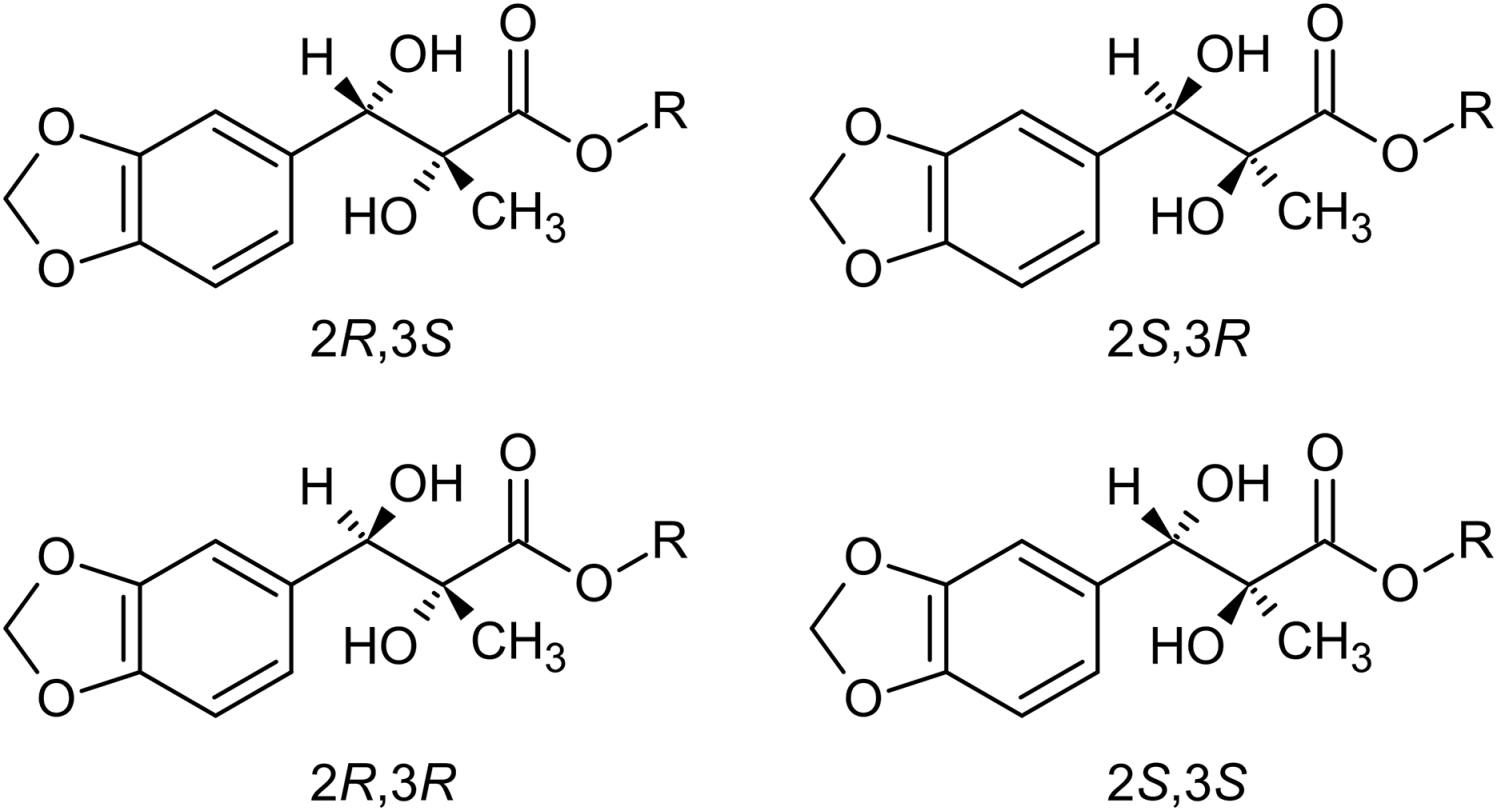
Stereoisomers of the alkyl‐3‐(1,3‐benzodioxol‐5‐yl)‐2,3‐dihydroxy‐2‐methylpropanoate compounds. R = CH_3_, CH_2_CH_3_.

Scheme 2 shows a proposed mechanism for the formation of the diol‐esters from the glycidate esters. First, the epoxide ring is protonated, which then opens after attack by a water molecule. Finally, deprotonation yields a diol‐ester. This type of reaction has been reported previously with acid‐catalysed ring‐opening of ethylene oxide [22] as well as with styrene‐oxide, which formed a vicinal diol (1‐phenyl‐1,2‐ethanediol) when heated at 60°C in water [23], consistent with the current work.

**SCHEME 2:**
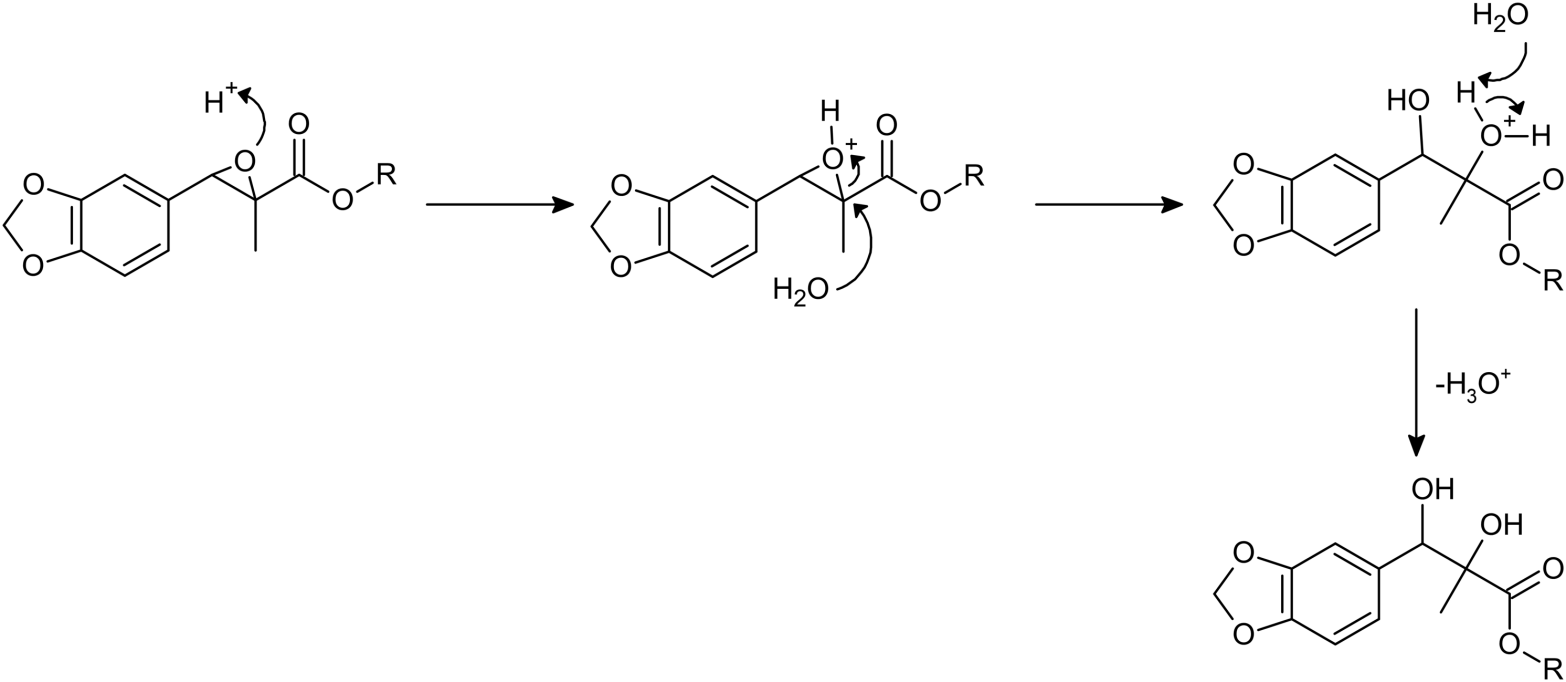
Proposed formation of alkyl‐3‐(1,3‐benzodioxol‐5‐yl)‐2,3‐dihydroxy‐2‐methylpropanoate.

Scheme 3 shows the formation of MDP2P from the diol‐ester intermediates. De‐esterification of the diol‐ester yields a diol‐carboxylic acid intermediate. Subsequent de‐carboxylation and dehydration of the benzylic carbon yield an enolate, which re‐arranges to give MDP2P. The benzylic carbon is more susceptible to protonation by acid as the corresponding carbocation is highly resonance stabilised by the methylenedioxyphenyl ring.

**SCHEME 3:**
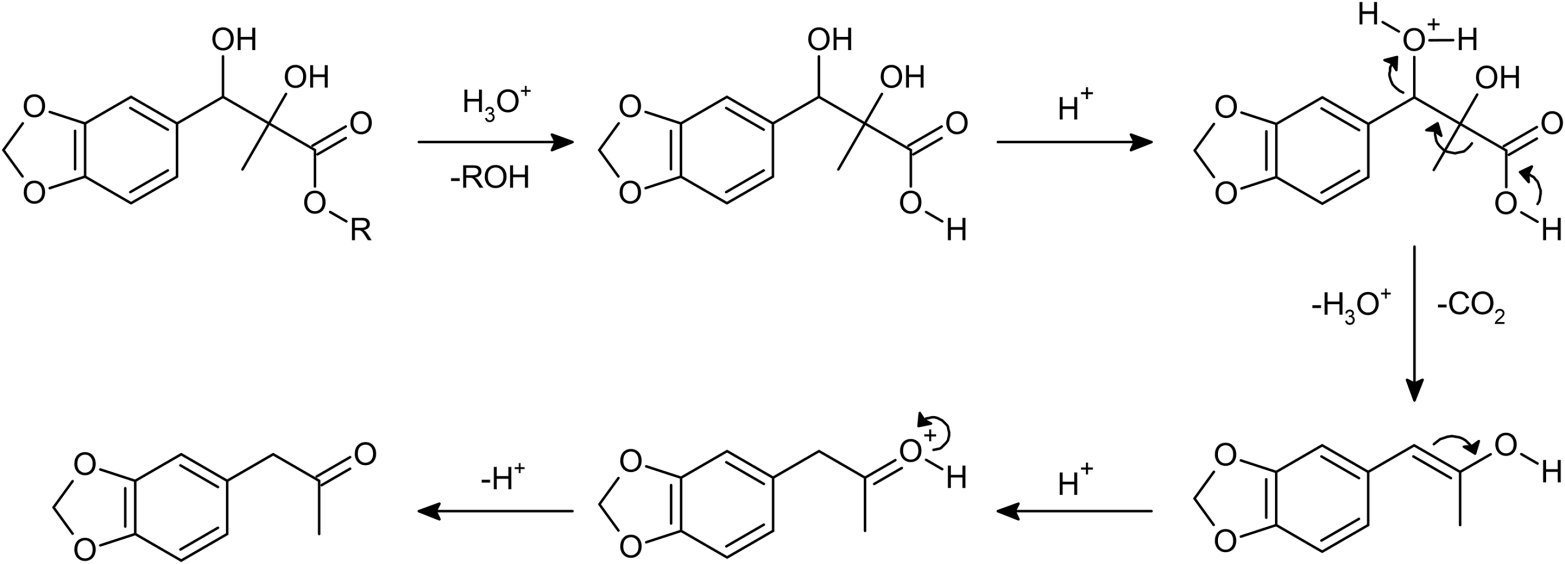
Proposed conversion of alkyl‐3‐(1,3‐benzodioxol‐5‐yl)‐2,3‐dihydroxy‐2‐methylpropanoate to MDP2P.

The new diol‐esters have not previously been reported and could be considered to be route‐specific impurities. Conversion to MDMA was not explored in this work; however, an investigation into whether the diol‐esters persist under reductive amination or Leuckart conditions would be of interest. No additional by‐products were detected in this work; however, larger scale synthesis as seen in clandestine operations may result in the formation of additional products.

#### 3.3.3 Effects of Reaction Time and Acid

Longer reaction times increased the yield of MDP2P from both MMDMG and EMDMG using hydrochloric acid (Entries 4–6 and 16–18, respectively, Table 3). The increase in yield between 6 and 18 h is negligible; however, the diol‐ester intermediate was detected after 6 h. Consequently, 18 h was sufficient to fully convert both MMDMG and EMDMG to MDP2P with hydrochloric acid resulting in yields of 79% and 75%, respectively.

Entries 7–12 for MMDMG and 19–25 (see Table 3) for EMDMG show the impact of the acid on the conversion of the glycidate esters to MDP2P. Sulphuric acid gave similar yields of MDP2P compared with hydrochloric acid. However, a black insoluble solid formed when heated at 100°C for 18 h with sulphuric acid, impacting the recovery of MDP2P. Sonication with water or dichloromethane did not break up the solid and hence possibly prevented the extraction of MDP2P. ^1^H NMR spectra of the reaction products contained signals from only MDP2P. Weaker acids, phosphoric acid and acetic acid, resulted in lower yields of MDP2P. Longer reaction times improved the yield of MDP2P with phosphoric acid (from 2% after 3 h to 29%–34% after 6 h); however, the major product was the diol‐ester. There was a small increase in the yield of MDP2P using acetic acid with longer reaction time from below limits of detection to 9%.

When MMDMG and EMDMG were heated at 100°C for 18 h in water only (Entry 2 for MMDMG and Entry 14 for EMDMG, Table 3), full conversion to the diol‐ester was observed indicating that the glycidate esters readily hydrolyse in water upon heating, and acid is not necessarily required for formation of the intermediate.

The reduced yield of MDP2P seen for phosphoric acid and acetic acid could be attributed to acid strength. Strong acids, such as hydrochloric acid and sulphuric acid are known to be more effective for de‐esterification due to increased reaction rates and higher concentrations of active protons. This could possibly explain why the diol‐ester is the major product of phosphoric acid and acetic acid reactions. The epoxide ring opening and hydration can take place (as seen in the absence of acid this still proceeds) but not de‐esterification due to lower concentrations of active protons and lower reaction rates. Longer reaction times may be required for full conversion to MDP2P using phosphoric acid and acetic acid; however, this was not explored in this work.

### 3.4 Base Hydrolysis (Method 2)

#### 3.4.1 Isolation of Sodium MDP2P Glycidate and Attempted Synthesis of MDP2P Glycidic Acid

Sodium MDP2P glycidate was synthesised from MMDMG or EMDMG using reported methods [9, 10]. The glycidate esters were heated in alcoholic sodium hydroxide for 5 h at reflux. Concentrated hydrochloric acid was then added in an attempt to isolate MDP2P glycidic acid. However, ^1^H NMR analysis indicated that the crude product was MDP2P. Multiple attempts were made to isolate MDP2P glycidic acid from sodium MDP2P glycidate with various amounts of hydrochloric acid, but each attempt resulted in MDP2P as the product. It was apparent that sodium MDP2P glycidate reacted rapidly with acid to form MDP2P. Thus, the reported procedure [9, 10] to reflux the reaction mixture for 18 h with copper powder is unnecessary. Other glycidic acids have been reported to be unstable under isolation conditions [5].

#### 3.4.2 Impact of Acid

Experimental data are presented in Table 4. As MMDMG and EMDMG both yield the same sodium MDP2P glycidate upon de‐esterification, EMDMG was used to explore the effect of different acids in one‐pot experiments. After de‐esterification, the resultant sodium MDP2P glycidate was stirred in aqueous acid for 10 min at room temperature. The yields of MDP2P obtained using hydrochloric acid, sulphuric acid and phosphoric acid are comparable to those of the acid hydrolysis methods (Method 1) with fair to good yields (~70%). The MDP2P produced was of high purity with negligible by‐products detected. Previous work suggested, based on experiments synthesising P2P, that 3,4‐methylenedioxylphenyl‐1‐propanone (MDP1P) may form as a by‐product during base hydrolysis [24]. This was not observed in the current work indicating that the methylenedioxy moiety influences the reactivity of the glycidate fragment. As observed in the acid hydrolysis experiments, acetic acid gave poor yields (~25%), although only MDP2P was observed in the ^1^H NMR spectrum of material extracted from the reaction mixture.

**TABLE 4:** Base hydrolysis (Method 2) reaction conditions and corresponding yields of MDP2P from MMDMG and EMDMG starting material.

Entry	Starting compound	Acid_(aq)_	Yield of MDP2P^a^
26	MMDMG	HCl	73%
27	EMDMG	HCl	71%
28	EMDMG	H_2_SO_4_	71%
29	EMDMG	H_3_PO_4_	69%
30	EMDMG	CH_3_COOH	25%

^a^ Average internal molar yield (%) calculated from GC–MS data (*n* = 3).

## 4 Conclusions

This work investigated the conversion of MMDMG and EMDMG to MDP2P via acid and base hydrolysis routes. Acid hydrolysis (Method 1) resulted in good yields of MDP2P (79% from MMDMG) after 18 h of heating at 100°C with hydrochloric acid. Time, temperature and the type of acid influenced the yields of MDP2P; higher temperatures, longer reaction times and stronger acids resulted in higher yields. The reactions proceed via diol‐ester intermediates, which have not previously been reported in literature. These compounds may be route‐specific impurities due to incomplete reaction or use of a weak acid such as acetic or phosphoric acid. The synthesised MDP2P was of a high purity with no by‐products detected. Base hydrolysis produced similar yields of MDP2P (73% with hydrochloric acid from MMDMG) with negligible by‐products detected. Although no additional by‐products were detected, laboratory experimental conditions can differ from those observed in clandestine operations, possibly causing differences in yield and by‐product formation.

Direct acid hydrolysis of glycidate esters is not common in the literature, rather, conversion first to sodium glycidate compounds via base hydrolysis is more general. However, these results demonstrate that acid hydrolysis (Method 1) affords slightly higher yields of MDP2P than the base/acid method (Method 2). The findings in this work contribute to the knowledge surrounding the evolving landscape of designer pre‐precursors. Understanding the factors that influence the yields of MP2P will aid forensic case work surrounding trends in illicit synthesis and assist in determining illicit drug yields from clandestine operations.

## Conflicts of Interest

The authors declare no conflicts of interest.

## Supporting information


**Figure S1:** EI mass spectrum of MMDMG (retention time = 9.989 min).
**Figure S2:** EI mass spectrum of EMDMG (retention time = 10.265 min).
**Figure S3:** EI mass spectrum of methyl‐3‐(1,3‐benzodioxol‐5‐yl)‐2,3‐dihydroxy‐2‐methylpropanoate (retention time = 10.392 min and 10.515 min).
**Figure S4:** EI mass spectrum of ethyl‐3‐(1,3‐benzodioxol‐5‐yl)‐2,3‐dihydroxy‐2‐methylpropanoate (retention time = 10.619 min and 10.734 min).
**Figure S5:** GC–MS total ion chromatogram of MMDMG for 3 h at room temperature (25°C) with no acid (control). MMDMG (9.989 min) and IS (13.636 min).
**Figure S6:** GC–MS total ion chromatogram of EMDMG for 3 h at room temperature (25°C) with no acid (control). EMDMG (10.265 min) and IS (13.635 min).
**Figure S7:** GC–MS total ion chromatogram of MMDMG for 18 h at 100°C with no acid (control). Methyl 3‐(1,3‐benzodioxol‐5‐yl)‐2,3‐dihydroxy‐2‐methylpropanoate (10.488 min) and IS (13.635 min).
**Figure S8:** GC–MS total ion chromatogram of EMDMG for 18 h at 100°C with no acid (control). Ethyl 3‐(1,3‐benzodioxol‐5‐yl)‐2,3‐dihydroxy‐2‐methylpropanoate (10.590 and 10.710 min) and IS (13.635 min).
**Figure S9:** GC–MS total ion chromatogram of MMDMG for 3 h at room temperature (25°C) with hydrochloric acid (Method 1). MMDMG (10.013 min), methyl 3‐(1,3‐benzodioxol‐5‐yl)‐2,3‐dihydroxy‐2‐methylpropanoate (10.392 and 10.515 min) and IS (13.681 min).
**Figure S10:** GC–MS total ion chromatogram of EMDMG for 3 h at room temperature (25°C) with hydrochloric acid (Method 1). EMDMG (10.292 min), ethyl 3‐(1,3‐benzodioxol‐5‐yl)‐2,3‐dihydroxy‐2‐methylpropanoate (10.619 and 10.737 min) and IS (13.681 min).
**Figure S11:** GC–MS total ion chromatogram of MMDMG for 3 h at 100°C with hydrochloric acid (Method 1). MDP2P (8.543 min), methyl 3‐(1,3‐benzodioxol‐5‐yl)‐2,3‐dihydroxy‐2‐methylpropanoate (10.392 and 10.515 min) and IS (13.681 min).
**Figure S12:** GC–MS total ion chromatogram of EMDMG for 3 h at 100°C with hydrochloric acid (Method 1). MDP2P (8.536 min), ethyl 3‐(1,3‐benzodioxol‐5‐yl)‐2,3‐dihydroxy‐2‐methylpropanoate (10.612 and 10.731 min) and IS (13.681 min).
**Figure S13:** GC–MS total ion chromatogram of MMDMG for 6 h at 100°C with hydrochloric acid (Method 1). MDP2P (8.533 min), methyl 3‐(1,3‐benzodioxol‐5‐yl)‐2,3‐dihydroxy‐2‐methylpropanoate (10.382 and 10.505 min) and IS (13.665 min).
**Figure S14:** GC–MS total ion chromatogram of EMDMG for 6 h at 100°C with hydrochloric acid (Method 1). MDP2P (8.532 min), ethyl 3‐(1,3‐benzodioxol‐5‐yl)‐2,3‐dihydroxy‐2‐methylpropanoate (10.607 and 10.726 min) and IS (13.665 min).
**Figure S15:** GC–MS total ion chromatogram of MMDMG for 18 h at 100°C with hydrochloric acid (Method 1). MDP2P (8.532 min) and IS (13.664 min).
**Figure S16:** GC–MS total ion chromatogram of EMDMG for 18 h at 100°C with hydrochloric acid (Method 1). MDP2P (8.532 min) and IS (13.664 min).
**Figure S17:** GC–MS total ion chromatogram of MMDMG for 3 h at 100°C with sulphuric acid (Method 1). MDP2P (8.521 min), methyl 3‐(1,3‐benzodioxol‐5‐yl)‐2,3‐dihydroxy‐2‐methylpropanoate (10.382 and 10.492 min) and IS (13.647 min).
**Figure S18:** GC–MS total ion chromatogram of EMDMG for 3 h at 100°C with sulphuric acid (Method 1). MDP2P (8.521 min), ethyl 3‐(1,3‐benzodioxol‐5‐yl)‐2,3‐dihydroxy‐2‐methylpropanoate (10.595 and 10.714 min) and IS (13.646 min).
**Figure S19:** GC–MS total ion chromatogram of MMDMG for 18 h at 100°C with sulphuric acid (Method 1). MDP2P (8.516 min) and IS (13.636 min).
**Figure S20:** GC–MS total ion chromatogram of EMDMG for 18 h at 100°C with sulphuric acid (Method 1). MDP2P (8.516 min) and IS (13.636 min).
**Figure S21:** GC–MS total ion chromatogram of MMDMG for 3 h at 100°C with phosphoric acid (Method 1). MDP2P (8.521 min), methyl 3‐(1,3‐benzodioxol‐5‐yl)‐2,3‐dihydroxy‐2‐methylpropanoate (10.369 and 10.493 min) and IS (13.646 min).
**Figure S22:** GC–MS total ion chromatogram of EMDMG for 3 h at 100°C with phosphoric acid (Method 1). MDP2P (8.521 min), ethyl 3‐(1,3‐benzodioxol‐5‐yl)‐2,3‐dihydroxy‐2‐methylpropanoate (10.596 and 10.716 min) and IS (13.647 min).
**Figure S23:** GC–MS total ion chromatogram of MMDMG for 18 h at 100°C with phosphoric acid (Method 1). MDP2P 8.512 min), methyl 3‐(1,3‐benzodioxol‐5‐yl)‐2,3‐dihydroxy‐2‐methylpropanoate (10.360 and 10.482 min) and IS (13.629 min).
**Figure S24:** GC–MS total ion chromatogram of EMDMG for 18 h at 100°C with phosphoric acid (Method 1). MDP2P (8.512 min), ethyl 3‐(1,3‐benzodioxol‐5‐yl)‐2,3‐dihydroxy‐2‐methylpropanoate (10.586 and 10.706 min) and IS (13.629 min).
**Figure S25:** GC–MS total ion chromatogram of MMDMG for 3 h at 100°C with acetic acid (Method 1). Methyl 3‐(1,3‐benzodioxol‐5‐yl)‐2,3‐dihydroxy‐2‐ methylpropanoate (10.369 and 10.493 min) and IS (13.647 min).
**Figure S26:** GC–MS total ion chromatogram of EMDMG for 3 h at 100°C with acetic acid (Method 1). Ethyl 3‐(1,3‐benzodioxol‐5‐yl)‐2,3‐dihydroxy‐2‐methylpropanoate (10.596 and 10.716 min) and IS (13.646 min).
**Figure S27:** GC–MS total ion chromatogram of MMDMG for 18 h at 100°C with acetic acid (Method 1). Methyl 3‐(1,3‐benzodioxol‐5‐yl)‐2,3‐dihydroxy‐2‐ methylpropanoate (10.359 and 10.483 min) and IS (13.630 min).
**Figure S28:** GC–MS total ion chromatogram of EMDMG for 18 h at 100°C with acetic acid (Method 1). MDP2P (8.512 min), ethyl 3‐(1,3‐benzodioxol‐5‐ yl)‐2,3‐dihydroxy‐2‐methylpropanoate (10.585 and 10.707 min) and IS (13.630 min).
**Figure S29:** GC–MS total ion chromatogram of sodium PMK glycidate (from MMDMG) for 10 min at 25°C with hydrochloric acid (Method 2). MDP2P (8.509 min) and IS (13.628 min).
**Figure S30:** GC–MS total ion chromatogram of sodium PMK glycidate (from EMDMG) for 10 min at 25°C with hydrochloric acid (Method 2). MDP2P (8.509 min) and IS (13.629 min).
**Figure S31:** GC–MS total ion chromatogram of sodium PMK glycidate (from EMDMG) for 10 min at 25°C with sulphuric acid (Method 2). MDP2P (8.509 min) and IS (13.628 min).
**Figure S32:** GC–MS total ion chromatogram of sodium PMK glycidate (from EMDMG) for 10 min at 25°C with phosphoric acid (Method 2). MDP2P (8.509 min) and IS (13.629 min).
**Figure S33:** GC–MS total ion chromatogram of sodium PMK glycidate (from EMDMG) for 10 min at 25°C with acetic acid (Method 2). MDP2P (8.509 min) and IS (13.628 min).
**Figure S34:** dta70143‐sup‐0001‐Supporting_Information.docx. ^1^H NMR spectrum of seized MMDMG starting material (CDCl_3_).
**Figure S35:** dta70143‐sup‐0001‐Supporting_Information.docx. ^13^C NMR spectrum of MMDMG starting material (CDCl_3_).
**Figure S36:** dta70143‐sup‐0001‐Supporting_Information.docx. ^1^H NMR spectrum of seized EMDMG starting material (CDCl_3_).
**Figure S37:** dta70143‐sup‐0001‐Supporting_Information.docx. ^13^C NMR spectrum of EMDMG starting material (CDCl_3_).
**Figure S38:** dta70143‐sup‐0001‐Supporting_Information.docx. ^1^H NMR spectrum of methyl‐3‐(1,3‐benzodioxol‐5‐yl)‐2,3‐dihydroxy‐2‐methylpropanoate (CDCl_3_).
**Figure S39:** dta70143‐sup‐0001‐Supporting_Information.docx. ^13^C NMR spectrum of methyl‐3‐(1,3‐benzodioxol‐5‐yl)‐2,3‐dihydroxy‐2‐methylpropanoate ((CD_3_)_2_SO).
**Figure S40:** dta70143‐sup‐0001‐Supporting_In‐formation.docx. ^1^H NMR spectrum of ethyl‐3‐(1,3‐benzodioxol‐5‐yl)‐2,3‐dihydroxy‐2‐methylpropanoate (CDCl_3_).
**Figure S41:** dta70143‐sup‐0001‐Supporting_Information.docx. ^13^C NMR spectrum of ethyl‐3‐(1,3‐benzodioxol‐5‐yl)‐2,3‐dihydroxy‐2‐methylpropanoate ((CD_3_)_2_SO).
**Figure S42:** dta70143‐sup‐0001‐Supporting_Information.docx. ^1^H NMR spectra (solvent: CDCl_3_) of methyl 3‐(1,3‐benzodioxol‐5‐yl)‐2,3‐dihydroxy‐2‐methylpropanoate (2.4–5 ppm region). Only the major isomer is annotated.
**Table S1:** Nuclear magnetic resonance spectroscopy parameters.
**Table S2:** Stability results for analytes after 1 week in storage (6°C).
**Table S3:** Recovery for selected analytes.

## Data Availability

The data that supports the findings of this study are available in the Supporting Information of this article.
