# Supplementary material for: An Investigation Into the Yields of 3,4‐Methylenedioxyphenyl‐2‐propanone (MDP2P) From Glycidate Pre‐Precursors
Source: Drug Test Anal. 2026 Sep 7;18(11):1470–9. doi: 10.1002/dta.70143 (PMC13727355; doi:10.1002/dta.70143)
Supplement: Supplementary file 1 [file DTA-18-1470-s001.docx]

**Supporting information**

**An Investigation into the Yields of 3,4-Methylenedioxyphenyl-2-propanone (MDP2P) from Glycidate Pre-Precursors**

Alexandra L. Mercieca^1^, Ella S. Schuett^1^, Andrew M. McDonagh^1^, Shanlin Fu^2^, Morgan Alonzo^1^*

^1^ School of Mathematical and Physical Sciences, University of Technology Sydney, Broadway NSW 2007 Australia

^2^ Centre for Forensic Science, School of Mathematical and Physical Sciences, University of Technology Sydney, Broadway NSW 2007 Australia

[*morgan.alonzo@uts.edu.au](mailto:*morgan.alonzo@uts.edu.au)

## NMR Spectroscopy Parameters

Table S1 Nuclear magnetic resonance spectroscopy parameters

| Parameter | ^1^H NMR | ^13^C NMR | qNMR |
| --- | --- | --- | --- |
| Frequency | 400.17 MHz | 100.63 MHz | 400.27 MHz |
| Spectral width | 8196.72 Hz | 23809.52 Hz | 8196.72 Hz |
| Relaxation delay | 1 s | 2 s | 45 s |
| Scans | 32 | 2048 | 64 |
| Acquisition time | 3.998 s | 1.376 s | 3.998 s |
| Pulse width | 30 ° | 30 ° | 30 ° |

*qNMR Procedure*

Deuterated dimethyl sulfoxide (DMSO-d_6_) was spiked with a known mass of the internal calibrant, benzoic acid (99.91% purity). Known quantities of MMDMG, EMDMG, MDP2P and the diol-esters were weighed using a 5 decimal place analytical balance (0.01 mg accuracy) and dissolved into 600 µL of the spiked DMSO-d_6_. The resulting solutions were then transferred to 5 mm NMR tubes.

## Synthesis and characterisation

*MDP2P*

MDP2P was prepared from 3,4-methylenedioxyphenylacetic acid using a literature procedure^1^ and vacuum distilled at 40 mbar and 185°C. The resultant yellow oil was purified using adapted literature procedures.^2^

**Purity** by qNMR = 95%. **^1^H NMR** 400 MHz, CDCl_3_ δ = 6.77 (d, *J =* 7.84 Hz, 1H), 6.67 (s, 1H), 6.64 (d, *J =* 7.92 Hz, 1H), 5.94 (s, 2H), 3.59 (s, 2H), 3.12 (s, 3H). **^13^C NMR** 400 MHz, CDCl_3_: δ = 29.26, 50.71, 101.19, 108.63, 109.89, 122.65, 127.95, 146.84, 148.06, 206.65. **MS** (**EI**, 75 eV) *m/z* 178 (M^+^, 25%), 135 (100), 77 (20), 51 (12), 136 (10).

*Alkyl-3-(1,3-benzodioxol-5-yl)-2,3-dihydroxy-2-methylpropanoate*

MMDMG (100 mg, 0.42 mmol) or EMDMG (100 mg, 0.40 mmol) was dissolved in water (5 mL) and refluxed in phosphoric acid (1.0 mL, 0.85 mmol) for 1 h. The reaction was then cooled and organic components extracted into dichloromethane (3 x 10 mL) and washed with water and brine. The organic portion was then dried over sodium sulfate, filtered and the solvent removed.

*Methyl-3-(1,3-benzodioxol-5-yl)-2,3-dihydroxy-2-methylpropanoate (2(S/R) and 3(S/R) isomers)*

Obtained as a white solid. **Yield:** 80% (by weight). **Purity** by qNMR = 95%. **TLC** (silica gel, hexane/ethyl acetate 7:3): *R*_f_ = 0.16. **^1^H NMR** 400 MHz, CDCl_3_. Major isomer: δ = 6.97 (d, *J* = 1.52 Hz, 1H), 6.78 (multiplet, overlapped, 2H), 5.95 (multiplet, overlapped, 2H), 4.75 (d, *J* = 6.44 Hz, 1H), 3.84 (s, 3H), 3.46 (s, 1H), 2.54 (d, *J* = 6.48 Hz, 1H), 1.19 (s, 3H). Minor isomer: δ = 6.78 (multiplet, overlapped, 3H), 5.95 (multiplet, overlapped, 2H), 4.67 (d, *J* = 7.60 Hz, 1H), 3.67 (s, 3H), 3.23 (s, 1H), 2.81 (d, *J* = 7.60 Hz, 1H), 1.54 (s, 3H).**^13^C NMR** 400 MHz, CDCl_3_: δ = 22.50, 53.31, 77.97, 101.25, 107.92, 108.35, 121.81, 132.75, 147.79, 147.81, 176.4973. (CD_3_)_2_SO: δ = 22.36, 51.78, 76.97, 77.38, 100.62, 106.86, 108.91, 121.66, 134.57, 146.29, 146.45, 175.76. **MS** (**EI**, 75 eV) *m/z* 254 (M^+^, 17%), 151 (100), 93 (50), 65 (24), 104 (18). **HRMS (ESI+)** *m/z*: [M + Na]^+^ calculated for C_12_H_14_O_6_Na, 277.0682; found, 277.0677, difference, -2.0 ppm.

*Ethyl-3-(1,3-benzodioxol-5-yl)-2,3-dihydroxy-2-methylpropanoate (2(S/R) and 3(S/R) isomers)*

Obtained as a colourless viscous oil. **Yield:** 83% (by weight). **Purity** by qNMR = 98%. **TLC** (silica gel, hexane/ethyl acetate 7:3): *R*_f_ = 0.19. **^1^H NMR** 400 MHz, CDCl_3_. Major isomer: δ = 6.97 (d, *J* = 1.60 Hz, 1H), 6.78 (multiplet, overlapped, 2H), 5.95 (multiplet, overlapped, 2H), 4.74 (d, *J* = 6.60 Hz, 1H), 4.30 (multiplet, 2H), 3.50 (s, 1H), 2.59 (d, *J* = 6.68 Hz, 1H), 1.33 (t, *J* = 7.12 Hz, 3H), 1.18 (s, 3H). Minor isomer: δ = 6.78 (multiplet, overlapped, 3H), 5.95 (multiplet, overlapped, 2H), 4.66 (d, *J* = 7.52 Hz, 1H), 4.10 (multiplet, 2H), 3.29 (s, 1H), 2.85 (d, *J* = 7.64 Hz, 1H), 1.54 (s, 3H), 1.20 (t, *J* = 8.04 Hz, 3H). **^13^C NMR** 400 MHz, CDCl_3_: δ = 14.30, 22.50, 62.52, 77.38, 77.93, 101.21, 107.87, 108.38, 121.81, 132.88, 147.74, 176.05. (CD_3_)_2_SO: δ = 14.13, 22.33, 60.37, 76.97, 77.23, 100.621, 106.86, 108.93, 121.65, 134.65, 146.28, 146.46, 175.25. **MS** (**EI**, 75 eV) *m/z* 268 (M^+^, 27%), 151 (100), 93 (48), 118 (32), 65 (26). **HRMS (ESI+)** *m/z*: [M + Na]^+^ calculated for C_13_H_16_O_6_Na, 291.0839; found, 291.0837, difference, -0.7 ppm.

## Method Validation Results

*Stability Analysis*

Table S2 Stability results for analytes after one week in storage (6°C)

| Analyte | Accuracy (MRE %) (*n*=3) | | | | Precision (%CV) (*n*=3) | | | |
| --- | --- | --- | --- | --- | --- | --- | --- | --- |
|  | Low QC | Med QC | High QC | Case sample | Low QC | Med QC | High QC | Case sample |
| MMDMG | 5.3 | -2.6 | -0.4 | -0.8 | 4.4 | 1.9 | 3.1 | 3.7 |
| EMDMG | 5.4 | -1.4 | 0.9 | -3.3 | 4.0 | 1.9 | 3.0 | 6.7 |
| MDP2P | 8.1 | -2.2 | -1.1 | -0.7 | 6.5 | 1.7 | 2.4 | 2.3 |

*Recovery*

Table S3 Recovery for selected analytes

| Analyte | Recovery (%) ± SD |
| --- | --- |
| MMDMG | 100 ± 4 |
| EMDMG | 98 ± 4 |
| MDP2P | 97 ± 0.1 |

*Polarimetry*

MMDMG: ${[\alpha]}_{D}^{20}-0.17$ (*c* 1.12, EtOAC)

EMDMG: ${[\alpha]}_{D}^{20}+0.62$ (*c* 1.75, EtOAC)

## GC-MS Mass Spectra

Figure S1 EI mass spectrum of MMDMG (retention time = 9.989 min).

Figure S2 EI mass spectrum of EMDMG (retention time = 10.265 min).

Figure S3 EI mass spectrum of methyl-3-(1,3-benzodioxol-5-yl)-2,3-dihydroxy-2-methylpropanoate (retention time = 10.392 min and 10.515 min).

Figure S4 EI mass spectrum of ethyl-3-(1,3-benzodioxol-5-yl)-2,3-dihydroxy-2-methylpropanoate (retention time = 10.619 min and 10.734 min).

## GC-MS Total Ion Chromatograms

Figure S5 GC-MS total ion chromatogram of MMDMG for 3 h at room temperature (25 °C) with no acid (control). MMDMG (9.989 min) and IS (13.636 min).

Figure S6 GC-MS total ion chromatogram of EMDMG for 3 h at room temperature (25 °C) with no acid (control). EMDMG (10.265 min) and IS (13.635 min).

Figure S7 GC-MS total ion chromatogram of MMDMG for 18 h at 100 °C with no acid (control). Methyl 3-(1,3-benzodioxol-5-yl)-2,3-dihydroxy-2-methylpropanoate (10.488 min) and IS (13.635 min).

Figure S8 GC-MS total ion chromatogram of EMDMG for 18 h at 100 °C with no acid (control). Ethyl 3-(1,3-benzodioxol-5-yl)-2,3-dihydroxy-2-methylpropanoate (10.590 and 10.710 min) and IS (13.635 min).

Figure S9 GC-MS total ion chromatogram of MMDMG for 3 h at room temperature (25 °C) with hydrochloric acid (Method 1). MMDMG (10.013 min), methyl 3-(1,3-benzodioxol-5-yl)-2,3-dihydroxy-2-methylpropanoate (10.392 and 10.515 min) and IS (13.681 min).

Figure S10 GC-MS total ion chromatogram of EMDMG for 3 h at room temperature (25 °C) with hydrochloric acid (Method 1). EMDMG (10.292 min), ethyl 3-(1,3-benzodioxol-5-yl)-2,3-dihydroxy-2-methylpropanoate (10.619 and 10.737 min) and IS (13.681 min).

Figure S11 GC-MS total ion chromatogram of MMDMG for 3 h at 100 °C with hydrochloric acid (Method 1). MDP2P (8.543 min), methyl 3-(1,3-benzodioxol-5-yl)-2,3-dihydroxy-2-methylpropanoate (10.392 and 10.515 min) and IS (13.681 min).

Figure S12 GC-MS total ion chromatogram of EMDMG for 3 h at 100 °C with hydrochloric acid (Method 1). MDP2P (8.536 min), ethyl 3-(1,3-benzodioxol-5-yl)-2,3-dihydroxy-2-methylpropanoate (10.612 and 10.731 min) and IS (13.681 min).

Figure S13 GC-MS total ion chromatogram of MMDMG for 6 h at 100 °C with hydrochloric acid (Method 1). MDP2P (8.533 min), methyl 3-(1,3-benzodioxol-5-yl)-2,3-dihydroxy-2-methylpropanoate (10.382 and 10.505 min) and IS (13.665 min).

Figure S14 GC-MS total ion chromatogram of EMDMG for 6 h at 100 °C with hydrochloric acid (Method 1). MDP2P (8.532 min), ethyl 3-(1,3-benzodioxol-5-yl)-2,3-dihydroxy-2-methylpropanoate (10.607 and 10.726 min) and IS (13.665 min).

Figure S15 GC-MS total ion chromatogram of MMDMG for 18 h at 100 °C with hydrochloric acid (Method 1). MDP2P (8.532 min) and IS (13.664 min).

Figure S16 GC-MS total ion chromatogram of EMDMG for 18 h at 100 °C with hydrochloric acid (Method 1). MDP2P (8.532 min) and IS (13.664 min).

Figure S17 GC-MS total ion chromatogram of MMDMG for 3 h at 100 °C with sulfuric acid (Method 1). MDP2P (8.521 min), methyl 3-(1,3-benzodioxol-5-yl)-2,3-dihydroxy-2-methylpropanoate (10.382 and 10.492 min) and IS (13.647 min).

Figure S18 GC-MS total ion chromatogram of EMDMG for 3 h at 100 °C with sulfuric acid (Method 1). MDP2P (8.521 min), ethyl 3-(1,3-benzodioxol-5-yl)-2,3-dihydroxy-2-methylpropanoate (10.595 and 10.714 min) and IS (13.646 min).

Figure S19 GC-MS total ion chromatogram of MMDMG for 18 h at 100 °C with sulfuric acid (Method 1). MDP2P (8.516 min) and IS (13.636 min).

Figure S20 GC-MS total ion chromatogram of EMDMG for 18 h at 100 °C with sulfuric acid (Method 1). MDP2P (8.516 min) and IS (13.636 min).

Figure S21 GC-MS total ion chromatogram of MMDMG for 3 h at 100 °C with phosphoric acid (Method 1). MDP2P (8.521 min), methyl 3-(1,3-benzodioxol-5-yl)-2,3-dihydroxy-2-methylpropanoate (10.369 and 10.493 min) and IS (13.646 min).

Figure S22 GC-MS total ion chromatogram of EMDMG for 3 h at 100 °C with phosphoric acid (Method 1). MDP2P (8.521 min), ethyl 3-(1,3-benzodioxol-5-yl)-2,3-dihydroxy-2-methylpropanoate (10.596 and 10.716 min) and IS (13.647 min).

Figure S23 GC-MS total ion chromatogram of MMDMG for 18 h at 100 °C with phosphoric acid (Method 1). MDP2P 8.512 min), methyl 3-(1,3-benzodioxol-5-yl)-2,3-dihydroxy-2-methylpropanoate (10.360 and 10.482 min) and IS (13.629 min).

Figure S24 GC-MS total ion chromatogram of EMDMG for 18 h at 100 °C with phosphoric acid (Method 1). MDP2P (8.512 min), ethyl 3-(1,3-benzodioxol-5-yl)-2,3-dihydroxy-2-methylpropanoate (10.586 and 10.706 min) and IS (13.629 min).

Figure S25 GC-MS total ion chromatogram of MMDMG for 3 h at 100 °C with acetic acid (Method 1). Methyl 3-(1,3-benzodioxol-5-yl)-2,3-dihydroxy-2-methylpropanoate (10.369 and 10.493 min) and IS (13.647 min).

Figure S26 GC-MS total ion chromatogram of EMDMG for 3 h at 100 °C with acetic acid (Method 1). Ethyl 3-(1,3-benzodioxol-5-yl)-2,3-dihydroxy-2-methylpropanoate (10.596 and 10.716 min) and IS (13.646 min).

Figure S27 GC-MS total ion chromatogram of MMDMG for 18 h at 100 °C with acetic acid (Method 1). Methyl 3-(1,3-benzodioxol-5-yl)-2,3-dihydroxy-2-methylpropanoate (10.359 and 10.483 min) and IS (13.630 min).

Figure S28 GC-MS total ion chromatogram of EMDMG for 18 h at 100 °C with acetic acid (Method 1). MDP2P (8.512 min), ethyl 3-(1,3-benzodioxol-5-yl)-2,3-dihydroxy-2-methylpropanoate (10.585 and 10.707 min) and IS (13.630 min).

Figure S29 GC-MS total ion chromatogram of sodium PMK glycidate (from MMDMG) for 10 min at 25 °C with hydrochloric acid (Method 2). MDP2P (8.509 min) and IS (13.628 min).

Figure S30 GC-MS total ion chromatogram of sodium PMK glycidate (from EMDMG) for 10 min at 25 °C with hydrochloric acid (Method 2). MDP2P (8.509 min) and IS (13.629 min).

Figure S31 GC-MS total ion chromatogram of sodium PMK glycidate (from EMDMG) for 10 min at 25 °C with sulfuric acid (Method 2). MDP2P (8.509 min) and IS (13.628 min).

Figure S32 GC-MS total ion chromatogram of sodium PMK glycidate (from EMDMG) for 10 min at 25 °C with phosphoric acid (Method 2). MDP2P (8.509 min) and IS (13.629 min).

Figure S33 GC-MS total ion chromatogram of sodium PMK glycidate (from EMDMG) for 10 min at 25 °C with acetic acid (Method 2). MDP2P (8.509 min) and IS (13.628 min).

## NMR Spectra

Figure S34 ^1^H NMR spectrum of seized MMDMG starting material (CDCl_3_).

Figure S35 ^13^C NMR spectrum of MMDMG starting material (CDCl_3_)

Figure S36 ^1^H NMR spectrum of seized EMDMG starting material (CDCl_3_).

Figure S37 ^13^C NMR spectrum of EMDMG starting material (CDCl_3_)

Figure S38 ^1^H NMR spectrum of methyl-3-(1,3-benzodioxol-5-yl)-2,3-dihydroxy-2-methylpropanoate (CDCl_3_).

Figure S39 ^13^C NMR spectrum of methyl-3-(1,3-benzodioxol-5-yl)-2,3-dihydroxy-2-methylpropanoate ((CD_3_)_2_SO).

Figure S40 ^1^H NMR spectrum of ethyl-3-(1,3-benzodioxol-5-yl)-2,3-dihydroxy-2-methylpropanoate (CDCl_3_).

Figure S41 ^13^C NMR spectrum of ethyl-3-(1,3-benzodioxol-5-yl)-2,3-dihydroxy-2-methylpropanoate ((CD_3_)_2_SO).

Figure S42 ^1^H NMR spectra (solvent: CDCl_3_) of methyl 3-(1,3-benzodioxol-5-yl)-2,3-dihydroxy-2-methylpropanoate (2.4 – 5 ppm region). Only the major isomer is annotated.

## References

1.	Tran K-V, Bickar D. Dakin− West synthesis of β-aryl ketones. *J Org Chem* 2006;71(17):6640-6643. doi: 10.1021/jo0607966.

2.	Lebedev A, Lebedeva A, Sheludyakov V, et al. Vilsmeier formylation of hydrazones and semicarbazones derived from alkyl, benzyl, and cycloalkyl methyl ketones. *Russ J Gen Chem* 2005;75:412-416. doi: 10.1007/s11176-005-0241-y.
